# Advances in MoS_2_-Based Field Effect Transistors (FETs)

**DOI:** 10.1007/s40820-015-0034-8

**Published:** 2015-02-13

**Authors:** Xin Tong, Eric Ashalley, Feng Lin, Handong Li, Zhiming M. Wang

**Affiliations:** 1 Institute of Fundamental and Frontier Sciences, University of Electronic Science and Technology of China, Chengdu, 610054 People’s Republic of China; 2 State Key Laboratory of Electronic Thin Films and Integrated Devices, University of Electronic Science and Technology of China, Chengdu, 610054 People’s Republic of China

**Keywords:** MoS_2_ FETs engineering, Low-frequency noise, Optical properties, MoS_2_ sensors, MoS_2_ memory devices

## Abstract

This paper reviews the original achievements and advances regarding the field effect transistor (FET) fabricated from one of the most studied transition metal dichalcogenides: two-dimensional MoS_2_. Not like graphene, which is highlighted by a gapless Dirac cone band structure, Monolayer MoS_2_ is featured with a 1.9 eV gapped direct energy band thus facilitates convenient electronic and/or optoelectronic modulation of its physical properties in FET structure. Indeed, many MoS_2_ devices based on FET architecture such as phototransistors, memory devices, and sensors have been studied and extraordinary properties such as excellent mobility, ON/OFF ratio, and sensitivity of these devices have been exhibited. However, further developments in FET device applications depend a lot on if novel physics would be involved in them. In this review, an overview on advances and developments in the MoS_2_-based FETs are presented. Engineering of MoS_2_-based FETs will be discussed in details for understanding contact physics, formation of gate dielectric, and doping strategies. Also reported are demonstrations of device behaviors such as low-frequency noise and photoresponse in MoS_2_-based FETs, which is crucial for developing electronic and optoelectronic devices.

## Introduction

TMDCs (MoSe_2_, MoTe_2_, WS_2_, and WSe_2_, etc.) are well studied layered materials with sizable bandgap, which can be changed from bulk to layered form (indirect to direct transition), thus resulting in unique physical properties that are expected to be employed in future semiconducting devices [1, 2]. In particular, molybdenum disulfide (MoS_2_), which is conventionally prepared by scotch tape technique and chemical vapor deposition (CVD) method, has been a subject of great interest for several decades due to its interesting electronic and optical properties in its layered form, nanostructure and other architectures [3–12]. Numerous studies worldwide have studied how to apply this promising material in next-generation electronic and optoelectronic devices such as resonators [13], phototransistors [14], chemical sensors [15], photodetectors [16], amplifiers [17], and batteries [18, 19]. Specially, controllable valley polarization of MoS_2_ layered material suggests its potential in valleytronic devices [20, 21]. Being an example of the simplest form of layered MoS_2_, monolayer MoS_2_ has been under intensive investigation, in contrast to graphene [22], another monolayer of carbon, which remains immature due to its gapless characteristic. Several research groups have also investigated nanostructures of MoS_2_ in fabricating MoS_2_ devices, including nanosheet and nanoribbon transistors [23–25]. Bandgap of MoS_2_ layered structure varies from 1.2 eV for indirect bandgap to 1.9 eV for direct bandgap [26], playing a critical role in the development of future semiconductor devices, esp. optoelectronic devices. Since the first investigation of single-layer MoS_2_-based transistor and MoS_2_-based FET structure has become an important issue in electronic and optoelectronic devices evolution, additional knowledge in this respect is necessary for enhancing the performance of MoS_2_-based FET in future electronic and optoelectronic devices.

## MoS_2_-Based FETs Engineering

### Contact Engineering

MoS_2_-based FET has been demonstrated to exhibit high ON/OFF ratio exceeding 10^8^, suggested hundreds of mobilities and low subthreshold swing at room temperature, indicating its potential employment in future electronic devices [27, 28]. However, due to the obstacle of contact resistance in achieving high-performance circuit [29], it is essential to study the contact engineering as well as intrinsic properties of MoS_2_-based FET to approach roadmap of prospective applications of MoS_2_ and other 2D TMDCs.

Choosing various contact metals with different work function is critical in fabricating MoS_2_-based FETs, low contact resistance is expected and able to form lower Schottky barrier at MoS_2_-metal interface, thus allowing high performance in MoS_2_-based FET [30, 31]. Utilizing low work function metal scandium (Sc) as contact metal have realized a low contact resistance and high carrier injection n-type MoS_2_-based FET, which is demonstrated to largely eliminate the effect of contact resistance, thus reaching relatively high mobility up to 700 cm^2^ (V s)^−1^ in a high-*k* dielectric environment (will be discussed later) [32].

Kaustav Banerjee et al. have proposed a comprehensive study of contact metals (In, Ti, and Mo) of MoS_2_-based FETs. Generally, carrier injection is suppressed due to the formation of tunnel barrier by 2D MoS_2_ at the MoS_2_-metal interface. Meanwhile, to date, no appropriate contact metal can form ohmic contact with MoS_2_, resulting in the formation of Schottky barrier at MoS_2_-metal interface. To reduce both the Schottky barrier and contact resistance of MoS_2_ FET, metal In performs well to some degree but creates a large tunnel barrier; in contrast, tunnel barrier is barely observed when using Pd as the contact metal to MoS_2_ [33]. Furthermore, employing metal Ti as contact metal can lead to a lower Schottky Barrier. However, it is still able to reduce the injection of electrons and the unstable properties of Ti also limit its high performance in MoS_2_ FETs [34].

**Fig. 1:**
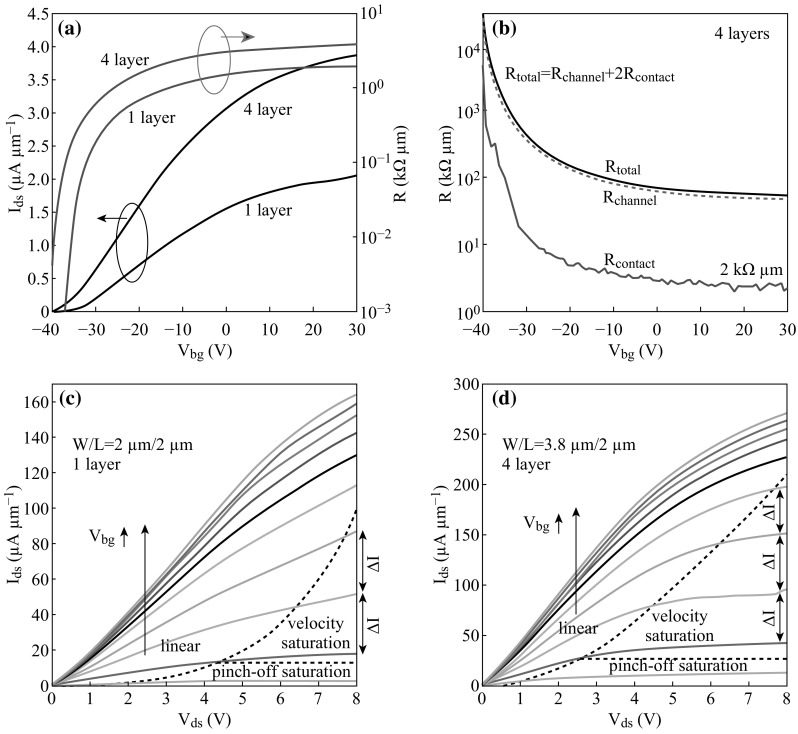
**a** *I*_ds_ (drain-source current) versus *V*_bg_ (back-gate voltage) curves for 1-layer and 4-layers MoS_2_ FETs(*V*_ds_ = 0.1 V). **b** Different *V*_bg_ and corresponding contact resistance (*R*_contact_), channel resistance (*R*_channel_) and total resistance (*R*_total_) of 4-layers MoS_2_ FET under the condition of *I*_ds_ = 0.1 µA. **c** and **d** illustrated the output characteristics(*I*_ds_ vs. *V*_ds_) of 1-layer and 4-layers MoS_2_ FET, respectively. Adopted from [34]

To overcome the difficulties mentioned above, Kaustav Banerjee et al. propose an effective method to utilize Mo as contact metal and fabricate Mo (10 nm)/Au (100 nm) source/drain contacts on the Al_2_O_3_/Si substrate to achieve 1-layer and 4-layers MoS_2_ FETs [35]. As illustrated in Fig. 1, the drain-source current (*I*_ds_) versus back-gate voltage (*V*_bg_) curves (blue for log scale, black for linear) for 1-layer and 4-layers MoS_2_ FETs are shown in Fig. 1a, exhibiting evident n-type property with ON/OFF ratio exceeding 10^3^ (under condition of 0.1 V drain-source voltage (*V*_ds_)). Figure 1b describes the *V*_bg_ (ranging from −40 to 30 V) and corresponding contact resistance (*R*_contact_), channel resistance (*R*_channel_), and total resistance (*R*_total_) of 4-layers MoS_2_ FET under the condition of *I*_ds_ = 0.1 µA. Compared with the contact resistance of Ti contact [36] (~80 kΩ µm) and Ni/Au contact [31] (~4.5 kΩ µm) MoS_2_ FET, the contact resistance of Mo contact MoS_2_ FET is much lower (~2 kΩ µm), manifesting more potential for high-performance digital circuit. In addition, Fig. 1c, d illustrates the output characteristics (*I*_ds_ vs. *V*_ds_) of 1-layer and 4-layers MoS_2_ FETs with an inconspicuous Schottky contact, the black arrow denotes the increasing *V*_bg_ (from −30 to 30 V). Moreover, pinch-off saturation is not available for these MoS_2_ FETs, but velocity saturation is suitable for use of as-fabricated device, which is suggested by Δ*I* in Fig. 1c, d. To summarize, Mo contact multilayer MoS_2_ FETs possess low contact resistances (~2 kΩ µm), high ON-currents (271 µA µm^−1^ at *V*_ds_ = 8 V), and reasonable mobilities (~27 cm^2^ (V s)^−1^), exhibiting more potential applications in high performance digital devices than monolayer MoS_2_ FETs.

**Fig. 2:**
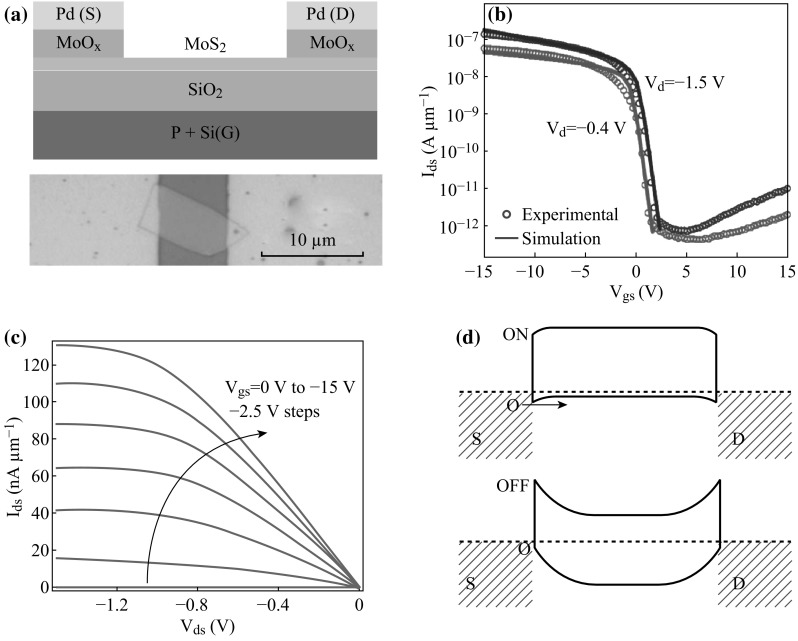
**a** Schematic architecture and optical image of as-fabricated FET. **b** *I*_ds_ versus *V*_gs_ characteristics, *V*_d_ in *red curve* (−0.4 V) and *blue curve* (−1.5 V) are measured, locus of *circle* and *solid line* denote experimental and simulated results, respectively. **c** *I*_ds_ versus *V*_ds_ characteristics and *V*_gs_ ranging from 0 to 15 V with a 2.5 V step are concerned. **d** Band diagram of as-fabricated p-type FET for the ON and OFF state. Adopted from [38]. (Color figure online)

For most of the contact metals, Fermi level pinning close to the conduction band of MoS_2_ leads to limitation of hole injection, further detrimentally impact the realization of high-performance p-type MoS_2_ FET. Marcio Fontana et al. have demonstrated that Pd contact metal was available to form p-type MoS_2_ three-contact devices [37]. However, it depends a lot on large gate fields of these devices, which facilitates the decrease of Schottky barrier height in external electric field. Herein, Steven Chuang et al. introduced MoO_*x*_ (*x*≦3) as contact metal fabricated on MoS_2_ FET, which exhibits p-type behavior, demonstrating that the MoO_*x*_ is an efficient hole injection layer to MoS_2_ [38]. As a high work function material (6.6 eV) [39], MoO_*x*_ is regarded as a promising candidate for hole injector of MoS_2_. In this experiment, Steven Chuang et al. fabricated 30 nm Pd/30 nm MoO_*x*_ contact on 260 nm SiO_2_/Si substrate and successfully achieve p-type MoS_2_ FET. The schematic architecture and optical image of as-fabricated FET are shown in Fig. 2a. Figure 2b exhibits *I*_ds_ versus gate-source voltages (*V*_gs_) characteristics, different drain voltages (*V*_d_) in red curve (−0.4 V) and blue curve (−1.5 V) are measured, and locus of circle and solid line denotes experimental and simulated results, respectively. Figure 2c shows *I*_ds_ versus *V*_ds_ characteristics and *V*_gs_ along the arrow varying from 0 to 15 V with a 2.5 V step are concerned. Figure 2d displays the band diagram of as-fabricated p-type FET for the ON/OFF state. The MoS_2_ FET with MoO_*x*_ contact is demonstrated presenting evident p-type characteristics with on-current (*I*_on_)/off-current (*I*_off_) ~10^4^, manifesting expected hole contact of MoO_*x*_ electrode to the valence band. More importantly, this work leads to the exploration in high work function materials employed as alternative metal contacts to realize high-performance MoS_2_-based FETs.

At the same time, MoS_2_-based FETs are also supposed to be employed into applications of spintronics, which are usually fabricated by ferromagnetic contacts, thus forming the MoS_2_/ferromagnetic interface. Again, Schottky barrier is demonstrated to exist at this interface [40], hindering the spin injection of electrons. To reduce the Schottky barrier and investigate future spin transport of MoS_2_-based devices, thin MgO layer, an additional tunnel barrier is utilized in single-layer MoS_2_ FET (Co contact), which results in the large decrease (about 84 %) of Schottky barrier [41]. Based on this investigation, Saroj Prasad Dash et al. further introduce TiO_2_ tunnel barrier in multilayer MoS_2_ FET (Co contact) to tune the contact resistance, which performs well and leads to a large increase of on-state current and mobility. Moreover, the channel conductance and magnetoresistance can be controlled by applying different gate voltages, which increase the possibilities for employment of MoS_2_ and other TMDCs for prospective applications of spintronics [42].

To study the intrinsic properties and estimate the quality of contact metals in MoS_2_-based FETs, four-terminal measurement is more important compared with two-terminal measurement [43, 44]. Toward this effort, N. R. Pradhan et al. fabricated the MoS_2_-based FET with approximate 20 layers MoS_2_, 300 nm silicon dioxide, and 8 Au contacts, and then, four-terminal measurement was used to study the intrinsic properties of MoS_2_-based FET, which could measure the effective mobility regardless of the impact of contact resistance at MoS_2_-metal interface. Compared with the past work by Peide D. Ye et al. [31], which was almost the same condition of as-fabricated FET, they found a large increase (~1 order of magnitude) of as-fabricated device in mobility (~300 vs. ~28 cm^2^ (V s)^−1^). In addition, Pablo Jarillo-Herrero et al. demonstrated that Hall measurement was able to nearly eliminate the contact resistance as well and Luis Balicas investigated another TMDC FET, i.e., the MoSe_2_ FET and found that the Hall mobilities (~250 cm^2^ (V s)^−1^) was higher than previously two-terminal measurement (~150–200 cm^2^ (V s)^−1^) [45, 46]. That is, four-terminal measurement is vital in investigating intrinsic properties of MoS_2_-based FETs and estimating the quality of contact metals. Future studies about MoS_2_-based FETs, even TMDCs devices should pay more attention to the four-terminal measurement.

In addition, Heung Cho Ko et al. utilized graphene as the electrodes for MoS_2_-based FET, which was also demonstrated to effectively reduce the Schottky barrier at MoS_2_/grapheme interface [47]. It is worth mentioning that Manish Chhowalla et al. proposed a novel method to reduce the contact resistance of MoS_2_-based FETs, they primarily considered two phases of MoS_2_: metallic 1T MoS_2_ and semiconducting 2H MoS_2_, later fabricated 1T MoS_2_ for electrodes and 2H MoS_2_ nanosheets for channel material in FET, and then, a very low contact resistance reaching 200–300 Ω µm was achieved under none gate bias, resulting in a high ON/OFF ratio exceeding 10^7^, subthreshold swing (95 mV/decade) and 85 μA μm^−1^ drive currents values [48].

### Dielectric Formation

To achieve high-performance MoS_2_-based FETs, the formation of high-*k* gate dielectric is important. For example, Madan Dubey et al. fabricated the MoS_2_ FET with and without a high-*k* Al_2_O_3_ dielectric, and then, measurements of mobilities indicated an increase of 6.0–16.1 cm^2^ (V s)^−1^ [49]. In studies of Saptarshi Das et al. which was discussed above, they similarly introduced the high-*k* Al_2_O_3_ dielectric, thus resulting in the increase of mobilities from 184 to 700 cm^2^ (V s)^−1^ [32]. Moreover, both theoretical and experimental studies show that high-*k* HfO_2_ dielectric is able to effectively enhance the performance of MoS_2_-based FETs [27, 50, 51]. High-*k* gate dielectric is suggested to reduce the Coulombic scattering, which improves the electronic properties of channel in MoS_2_-based FETs [52].

Generally, considering the uniformity and controllable thickness of the material to deposit, atomic layer deposition (ALD) technology is an effective method to deposit high-*k* gate dielectric. However, high-quality gate dielectric is difficult to deposit on 2D MoS_2_ by ALD, which attributes to the absence of dangling bonds and other active elements at the surface. Toward this effort, Peide D. Ye et al. investigated the deposition of high-*k* Al_2_O_3_ on MoS_2_ by ALD; they utilized water and trimethylaluminum (TMA) as precursor and lowered the temperature of substrate down to 200 °C, which successfully resulted in the formation of 10 nm uniform Al_2_O_3_ dielectric on MoS_2_ by physical adsorption [53]. However, reaction of precursors in low temperature could further lead to the impurities resided in as-deposited high-*k* film, which limited its electronic properties [54]. To overcome it, Hyoungsub Kim et al. introduced oxygen plasma treatment in deposition of Al_2_O_3_ and HfO_2_ on multilayer MoS_2_ by the same method of ALD; they used X-ray photoelectron spectroscopy (XPS) analysis and found that oxygen plasma-treated MoS_2_ formed Mo-oxide layer at its surface, which is demonstrated to improve the quality of as-grown high-*k* Al_2_O_3_ and HfO_2_ dielectric. This work indicates the promising of plasma-treated ALD method in formation of high-*k* gate dielectric on MoS_2_-based FET [55].

Deposition of high-*k* Al_2_O_3_ on ultraviolet-ozone (UV-O_3_)-treated MoS_2_ has also been studied. Uniform high-*k* Al_2_O_3_ film was achieved due to the removal of contaminations and the formation of slight S–O bonds at the MoS_2_ surface. It is necessary to mention that UV-O_3_ exposure did not break the Mo–S bonds and was a non-disruptive method to achieve high-quality Al_2_O_3_ dielectric deposition. The surface of UV-O_3_-treated MoS_2_ is also demonstrated to be a suitable layer for controllable deposition of uniform and ultrathin Al_2_O_3_ (~4 nm), which is more practical in MoS_2_-based FET technology [56]. In addition, Lanxia Cheng et al. investigated the ALD deposition of Al_2_O_3_ dielectric on MoS_2_ by precursors of TMA/H_2_O and TMA/O_3_ and studied the properties of two types of as-deposited thin films. They claimed that O_3_ was an important factor in high-quality ALD deposition, which resulted in the deposition of uniform, lower thickness (~5 nm) of dielectric layer without the S–O bonds generation at MoS_2_ surface and the improvement of growth rate [57].

The formation of high-*k* dielectric is critical for MoS_2_-based FETs technology, and the nature of no dangling bonds at the surface of 2D MoS_2_ allows discovering suitable precursors and pretreatments for ALD deposition, which is relatively effective in gate dielectric deposition. Lowering the temperature of substrate to achieve physical adsorption can lead to uniform high-*k* dielectric layer deposition, but it is limited by essential clean surface of MoS_2_ and hard to control the parameters of deposition. Oxygen plasma-treated MoS_2_ surface is also demonstrated to form high quality, uniform dielectric layer, but this method is regarded as destructive. These two methods are not very practical in MoS_2_-based FETs as the formation of uniformity layer can be achieved only when the thickness is enough (about 10 nm), which limit the scaling down of FET. In contrast, UV-O_3_ exposure and O_3_ precursor are non-destructive and able to deposit ultrathin dielectric layer (~5 nm), which are expected in gate dielectric formation of MoS_2_-based FETs and other TMDCs-based FETs.

### Doping Strategies

Appropriate doping is another effective method to achieve high-performance MoS_2_-based FETs, which is demonstrated to strongly affect the contact resistance of MoS_2_ FET instead of utilizing different contact metals, such as n-type doping from polyethyleneimine (PEI) molecules on multilayer MoS_2_-based FET [58]. Ultrathin MoS_2_ limits the doping methods (ion implantation, etc.) employed in other semiconductors, leading to the exploration of novel doping methods in MoS_2_-based FET technology.

**Fig. 3:**
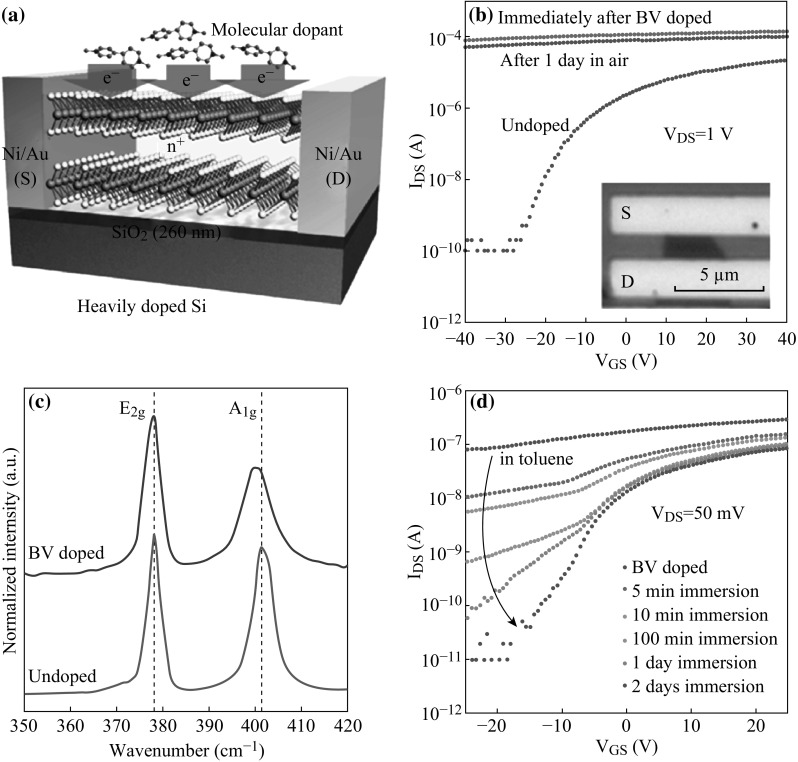
**a** Schematic diagram of BV doping on trilayer MoS_2_ FET. **b** Transfer characteristics of as-fabricated MoS_2_-based FET with and without doping. **c** Raman spectroscopy measurement. **d** Transfer characteristics of as-fabricated MoS_2_-based FET with different time in toluene. Adopted from [61]

Cesium Carbonate (Cs_2_CO_3_) has been employed to dope monolayer MoS_2_ FET [59], resulting in stable n-type doping and largely enhance the electron concentration in monolayer MoS_2_ (about 1 order of magnitude). Potassium has also been demonstrated to achieve degenerate n-doping of MoS_2_ FET in vacuum, indicating the essential of degenerate doping in high-performance MoS_2_ FET [60]. However, the unstable nature of Potassium limits its practical application. Herein, Daisuke Kiriya et al. proposed a doping strategy based on benzyl viologen (BV) [61], as illustrated in Fig. 3. Figure 3a depicts the schematic diagram of BV doping on trilayer MoS_2_ FET and the as-fabricated FET was put into the BV solution for 12-h doping; transfer characteristics of as-fabricated MoS_2_-based FET with and without doping are compared and shown in Fig. 3b. Before BV doping, the ON/OFF of MoS_2_ FET mainly depends on *V*_gs_ (ranging from −40 to 40 V) and the *I*_ds_ is about 2 × 10^−5^ A (*V*_gs_ = 40 V), following that BV doping indicates the less dependence of *V*_gs_ and the increase of *I*_ds_, which demonstrates the effective doping of BV method. Raman spectroscopy measurement is shown in Fig. 3c, depicting a red shift and realization of high electron density. Moreover, Fig. 3d shows that the as-doped FET is put into toluene and corresponding transfer characteristics with different time, which is promising for tuning the dopants density. This work represents an effective n-type doping method, which also reduces the contact resistance and improves the performance of MoS_2_-based FET.

Elements from halogen family are promising in doping MoS_2_. Toward this effort, chloride (Cl) molecular has been utilized to dope MoS_2_ FET by Lingming Yang et al. [62]. Few layer MoS_2_ is immersed into 1, 2 dichloroethane (DCE) over 12 h and then fabricated in MoS_2_ FET; n-type doping is elucidated by change of Fermi level, which is measured by XPS at the surface before and after doping. More importantly, the contact resistance of as-fabricated FET is reduced to a very low value (0.5 kΩ µm) after Cl molecular doping, thus resulting in a high drain current (460 mA mm^−1^). In addition, WS_2_ FET is also doped by the same method and even more effective than MoS_2_ FET, indicating that the Cl molecular doping is available in other TMDCs.

Doping strategies are significant in MoS_2_-based FETs and other TMDCs-based devices, and proper doping methods are expected in FET fabricated from ultrathin semiconducting materials. Note that, doping methods of 2D materials are still immature; studies are essential to explore stable, effective, and controllable doping strategies, which are practical and convenient in future nanoelectronic and optoelectronic devices.

## Low-Frequency Noise (LFN) Analysis in MoS_2_-Based FETs

The low-frequency noise (LFN) has been demonstrated as a limiting factor in high-performance electronic devices [63] and is generally called 1/*f* noise or flicker noise, which is first discovered in 1925 [64]. LFN determines the minimum value of signal level in electronic devices and circuits, affecting the realization of scaling down and lower power consumption in future circuits [65, 66], showing that it is necessary to study the LFN in MoS_2_-based FETs as this 2D layered material has been widely utilized for the fabrication of ultrascaled FET [67] and integrated circuits [68]. Analysis of LFN (measure the fluctuations of mobility conductivity or fluctuation of FET channel induced by carrier trapping or de-trapping) can help evaluate the quality of MoS_2_ FET [69]. Furthermore, for practical usage of MoS_2_ FET analog and digital electronic devices, it is necessary to reach the minimum requirement of LFN [70, 71].

**Fig. 4:**
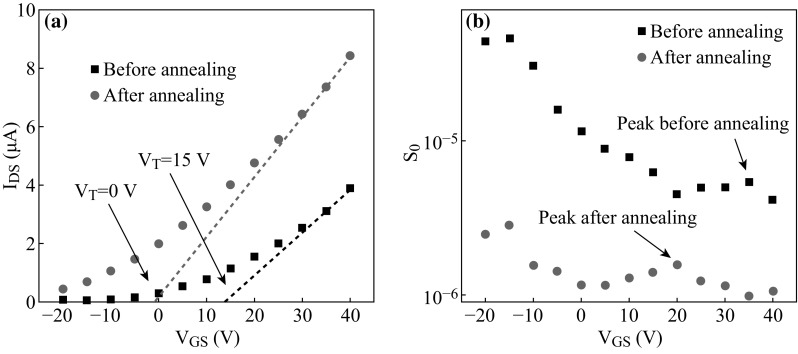
**a** *I*_ds_ versus *V*_gs_ characteristics at *V*_ds_ = 3 V of this FET, the *red* locus of points represents the curve after annealing (*V*_T_ = 0 V) and *black* locus of points represents the curve before annealing (*V*_T_ = 15 V). **b** The noise measurements before and after annealing at *V*_ds_ = 3 V. Adopted from [72]. (Color figure online)

Toward this effort, the LFN of bilayer MoS_2_ FET has been studied in details by Xie et al. [72]. The MoS_2_ FET is fabricated by a 1.2-nm-thick MoS_2_ (bilayer) thin film on a 300 nm SiO_2_/highly doped n-type Si substrate with 30-nm Ti/100-nm Au film as electrodes. The corresponding noise characteristics are measured and a new model of understanding the LFN in bilayer MoS_2_ FET was proposed. Different from 3D materials, the results exhibit a longer trap decay time in 2D materials with van der Waals bond (MoS_2_). Based on this model, an annealing is processed toward this bilayer MoS_2_ FET. Figure 4a shows the *I*_ds_ versus *V*_gs_ characteristics (*V*_gs_ ranging from −20 to 40 V) at *V*_ds_ = 3 V of this FET, the red locus of points represents the curve after annealing under condition of no threshold voltage (*V*_T_), and black locus of points represents the curve before annealing(*V*_T_ = 15 V), respectively. Figure 4b exhibits the noise measurements (*V*_gs_ ranging from −20 to 40 V) before and after annealing at *V*_ds_ = 3 V. A remarkable movement of noise peak to lower *V*_gs_ after annealing was observed, indicating that the decrease of trap density (annealing process) can effectively reduce the LFN in as-fabricated MoS_2_ FET.

**Fig. 5:**
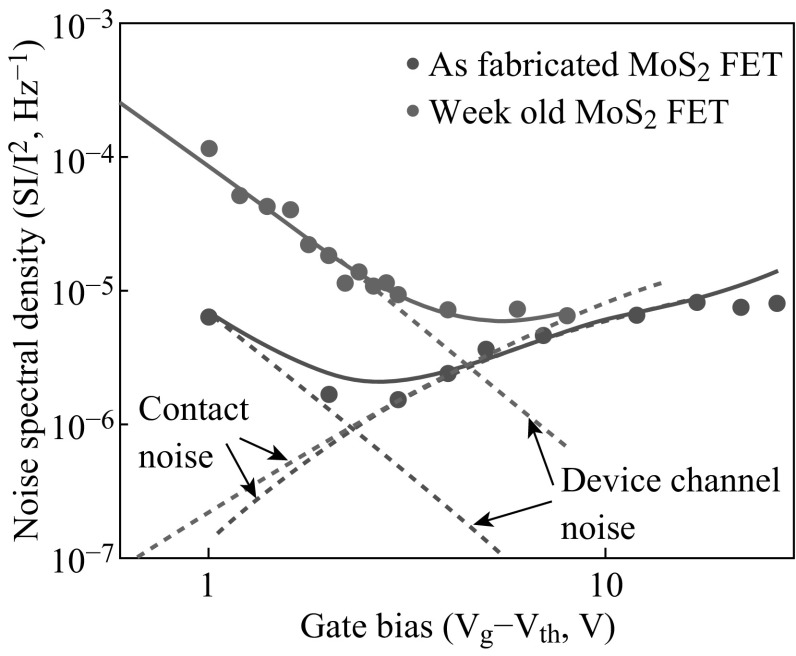
Noise spectral density as a function of gate bias before and after aging in as-fabricated MoS_2_ device. Adopted from [75]

While 1/*f* noise has been investigated in monolayer [73] and bilayer MoS_2_ FET, study of LFN in multilayer MoS_2_ FET is essential for its optimization. In order to achieve these goals, Kwon et al. [74] have investigated the LFN in multilayer MoS_2_ FET, which is architectured with a 40–50-nm MoS_2_ thin-film channel layer, 10 nm Ti/300 nm Au contact, and SiO_2_ on p-type silicon substrate. They studied the LFN behavior 1/*f* ^*γ*^ of as-fabricated MoS_2_ FET, where the *γ* is an exponent associated with distribution of traps. With the increase of gate voltage (*V*_G_), the trap in as-fabricated FET will be filled and *γ* will decrease and be stable at a value of 0.95. In contrast to the dominance of mobility fluctuation noise mechanism in monolayer MoS_2_ FET [73], the dominance of noise mechanism in as-fabricated multilayer MoS_2_ FET is demonstrated to be the carrier number fluctuation. Moreover, they found LFN characteristics of multilayer MoS_2_ FET are better than monolayer MoS_2_ FET, which attributes to its lower Hooge parameter related to the level of LFN. In addition, Renteria et al. [75] studied the relative contribution of channel and contact for LFN in multilayer MoS_2_ FET and demonstrated that the main mechanism of LFN is carrier number fluctuation, as depicted by Kwon et al. Moreover, they proposed a comparison of as-fabricated multilayer MoS_2_ FETs before and after aging. Figure 5 shows the noise spectral density before and after aging. It has been observed that the channel noise of MoS_2_ FET increased more than one order of magnitude after aging, but the increase of contact noise is very few. Thus, the phenomenon is mainly caused by the aging of the MoS_2_ channel rather than the aging of contact. This new phenomenon can be utilized in MoS_2_-based FET and other TMDCs- based FETs in terms of the optimization in channel implementation.

In addition, MoS_2_-metal contacts of MoS_2_-based FETs are also demonstrated to impact LFN [76]. The vacuum annealing strongly increases the transparency of contacts in FET, thus resulting the decrease of LFN. To conclude, LFN has been investigated in MoS_2_-based FETs, indicating the related factors are trap density, channel, and contact, which should be concerned for future circuits based on MoS_2_ and other 2D TMDCs.

## Optical Properties of MoS_2_-Based FETs

**Fig. 6:**
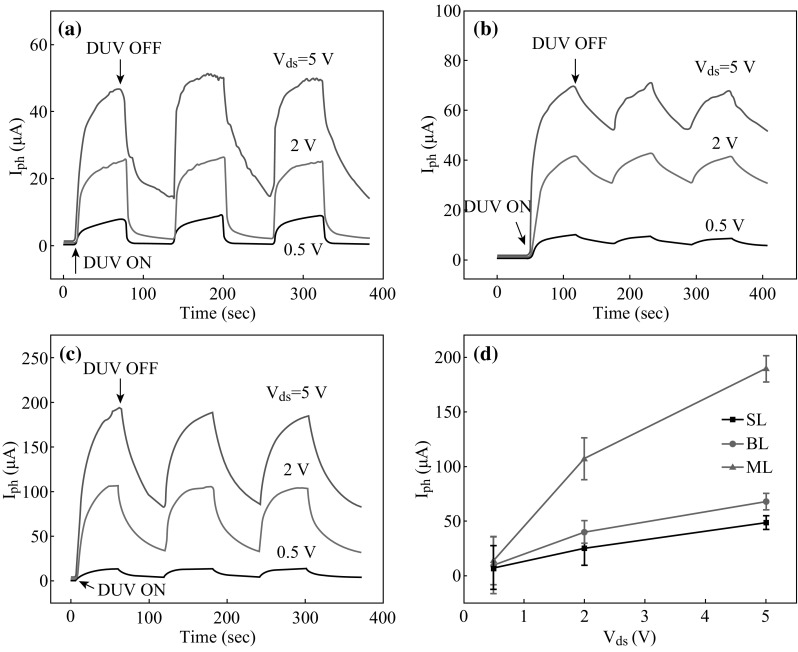
**a**−**c** Photocurrent (*I*_ph_) of monolayer, bilayer and multilayer MoS_2_ FETs measured in air (under the condition of *V*_ds_ = 0.5, 2.0, 5.0 V and *V*_G_ = 0), respectively. **d** Relative *I*_ph_ as a function of *V*_ds_ (0–5 V). Adopted from [80]

TMDCs have been widely fabricated in P–N junction devices, heterostructures, and phototransistors due to the outstanding photovoltaic effect [2, 77, 78]. Particularly, MoS_2_-based FETs have already been demonstrated to show a strong photoresponse [79]. To comprehensive study the optical properties of MoS_2_-based FETs, the number of MoS_2_ layers is concerned; herein, Jonghwa Eom et al. investigated layer-dependent MoS_2_ FETs (monolayer, bilayer, and multilayer) and measured the photocurrent response under different *V*_ds_ by using a 220 nm deep ultraviolet (DUV) light [80]. In Fig. 6, photocurrent (*I*_ph_) of monolayer, bilayer, and multilayer MoS_2_ FETs was measured in air (under the condition of *V*_ds_ = 0.5, 2.0, 5.0 V and V_G_ = 0) and illustrated in Fig. 6a–c, respectively. Figure 6d summarizes the results from Fig. 6a–c as a function of *V*_ds_ (0–5 V). They observed that monolayer and bilayer MoS_2_ FET exhibited a smaller value of photocurrent than multilayer MoS_2_ FET, which mainly attributed to a narrower bandgap and higher density of states in multilayer MoS_2_ FET. After turning off the light, relaxation time of photocurrent response was also measured in monolayer, bilayer, and multilayer MoS_2_ FETs; again, the smaller bandgap of multilayer MoS_2_ resulted in a shorter relaxation time in multilayer MoS_2_ FET. This work suggested that multilayer MoS_2_ FET was more promising than few layer MoS_2_ FET in photovoltaic applications, and as discussed above, multilayer MoS_2_-based FET with graphene electrode not only reduced the Schottky barrier height at MoS_2_/grapheme interface, but shows a 74 % optical transmittance (wavelength ranging from 400 to 800 nm), which is promising for transparent devices [47].

**Fig. 7:**
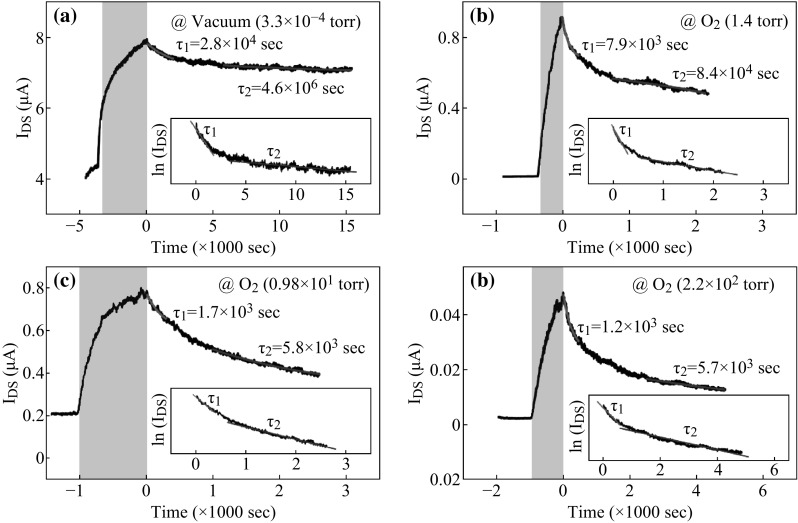
Photocurrent measurement under **a** 3.3 × 10^−4^ Torr vacuum condition, the shadow region means the UV light is on **b**–**d** shows the measurements under oxygen condition of 1.4 Torr, 0.98 × 10^1^ Torr, and 2.2 × 10^2^ Torr, respectively. Adopted from [81]

The photocurrent of MoS_2_ FET is always a significant topic to discuss. Cho et al. [81] have studied the decay of photocurrent in MoS_2_ FET; they fabricated the multilayer MoS_2_ nanosheet FETs and measured the decrease of photocurrent before and after turning off the UV light. Figure 7 illustrates the photocurrent measurements at different atmosphere in the same *V*_ds_ (0.1 V), and two constant of decay time *τ*_1_ and *τ*_2_ are also shown in Fig. 7. Figure 7a exhibits the photocurrent measurement under 3.3 × 10^−4^ Torr vacuum condition (shadow region represents on-state UV light). Figure 7b–d exhibits the measurements under oxygen condition of 1.4 Torr, 0.98 × 10^1^ Torr, and 2.2 × 10^2^ Torr. With the increase of oxygen pressure, photocurrent decreases faster, which is attributed to the charge trapping at the associated oxygen defect sites on MoS_2_ surface. In addition, they measured the decrease of photocurrent under different gate-bias stresses and found that when the gate-bias stress was negative, the decrease of photocurrent became slower and vice versa. Further study revealed that this phenomenon was caused by the increase of charge trapping (oxygen site) on MoS_2_ interface as well [82]. Moreover, resonant plasmonic nanoshells have also been deposited to fabricate MoS_2_ FET, which is demonstrated to be capable for the enhancement of photocurrent and photoluminescence [83].

As discussed above, regarding the transient time constant, isolated MoS_2_ FET manifested its potential in optoelectronics, which could reach magnitude of millisecond. However, Feng Wang et al. have investigated the optical properties of MoS_2_-WS_2_ heterostructure and the ultrafast dynamics of hole transfer and found a remarkable rise time shorter than 50 fs, which is demonstrated to hold large promising in future optoelectronic applications [84]. Based on this novel investigation, Su-Huai Wei et al. fabricated MoS_2_-WS_2_ heterostructure-based FET, which was demonstrated to possess high ON/OFF ratio exceeding 10^5^ and high photoresponsivity reaching 1.42 A W^−1^ [85].

The photoresponse of MoS_2_-based FETs shows promising for prospective applications of optoelectronics, compared with few layer MoS_2_; multilayer MoS_2_ is demonstrated to manifest better performance in photocurrent generation. Moreover, plasma-treated, novel nanostructured, and heterostuctured MoS_2_ are expected to fabricate high-performance MoS_2_-based FETs.

## MoS_2_-Based FETs Applications

### Applications of MoS_2_-Based FETs in Sensors

Due to the planar, atomic thin structure, possibility of large scale preparation, high surface-to-volume ratio and suggested bandgap, MoS_2_-based FET has been studied in sensor applications. Toward this effort, high-sensitivity pH sensor with reasonable range (3–9) and selectivity biosensor for protein detection (available at 100 femtomolar concentration) have been achieved by MoS_2_-based FET [86]. Similarly, for label-free biosensors, MoS_2_ nanosheet is promising and fabricated in FET, which exhibits high sensitivity in detecting cancer biomaker [87]. The as-fabricated FET is employed in liquid phase to selectively detect prostate-specific antigen (PSA) (cancer biomaker) by the change of drain current. That is, this method is potential for facilitating the development of cancer diagnostics in earlier time.

**Fig. 8:**
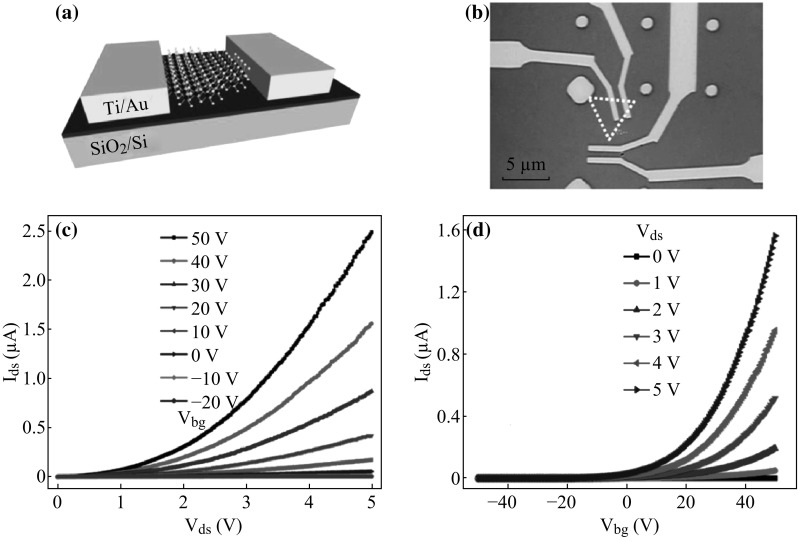
**a** Schematic diagram and **b** Optical image of the MoS_2_ FETs. **c** Output characteristics (*I*_ds_ vs. *V*_ds_) and **d** Transfer characteristics (*I*_ds_ vs. *V*_bg_) of the MoS_2_ FETs. Adopted from [88]

For gas sensor, Liu et al. [88] have focused on the Schottky-contacted CVD grown monolayer MoS_2_ FET. They fabricated the MoS_2_ FETs with 5 nm Ti/50 nm Au metal contact; the schematic diagram and optical image of MoS_2_ FETs are shown in Fig. 8a, b, respectively. Figure 8c exhibits the *I*_ds_ versus *V*_ds_ output characteristics of as-fabricated FET, and transfer characteristics (*I*_ds_ vs. *V*_bg_) is illustrated in Fig. 8d, manifesting the n-type characteristic, which corresponds to the n-type electronic property of MoS_2_ semiconductor [89]. Note that, there exists a Schottky barrier (SB) in as-fabricated MoS_2_ FET-based sensor (Fig. 8c). Later, they investigated the sensitivity and the mechanism of as-fabricated FET for detecting two poisonous gases: NO_2_ and NH_3_. Generally, conductance (resistance) change is measured to reflect performance of sensing and total resistance of as-fabricated FET is expressed as follows:

1 $$ R = R_{\text{channel}} + R_{\text{contact}} $$

In this equation, *R*_channel_ is only related to carrier concentration, but *R*_contact_ is related to both carrier concentration and the Schottky barrier height. The relationship is exponential, thus indicating that the Schottky barrier is a key factor in sensitivity. With a conductance change larger than 20 and 40 %, the sensitivity of this MoS_2_ FET-based chemical sensor can reach 20 parts per billion (ppb) for NO_2_ and 1 parts per million (ppm) for NH_3_, respectively. This detection limit is the lowest gas concentration detected compared with the other experiments: Li et al. [90] used multilayer MoS_2_ film FET to detect NO (detection limit ~800 ppb) and Late et al. [91] presented a detection limit of several hundred ppm for both NH_3_ and NO_2_ using atomically thin-layered MoS_2_ transistors. Moreover, Liu et al. found part of MoS_2_ devices exhibiting more Ohmic contact, but the little conductance change (<5 %) upon exposure to NO_2_ at concentrations up to 400 ppb further manifests that Schottky barrier modulation plays a more important role in these MoS_2_ FET-based sensors. That is, it is realizable to modulate the Schottky barrier contact of MoS_2_-based FET sensor and achieve higher performance at the sub-ppb level.

**Fig. 9:**
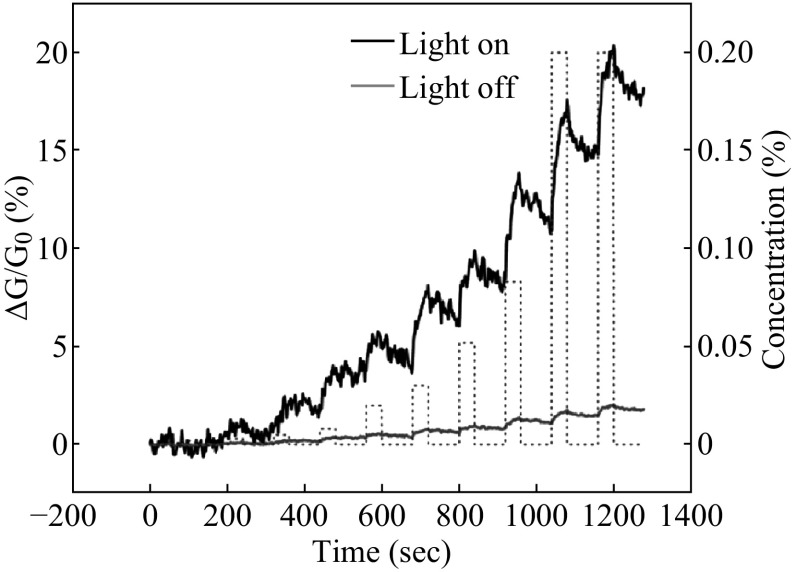
Sensitivity of triethylamine (TEA) with and without illumination. *Black line* represents on-state light and *red line* for off state. Adopted from [92]. (Color figure online)

Moreover, in monolayer MoS_2_-based FET sensor, sensitivity of detecting triethylamine (TEA) can be enhanced by illumination [92] as illustrated in Fig. 9. Δ*G* stands for the decrease of initial conductance (*G*_0_) of FET when exposed to TEA, the calculated Δ*G*/*G*_0_ is the sensitivity. The black line represents that the light is on and red line for off state, showing the increase of sensitivity with longer time (about 1 order of magnitude), which may attributes to the enhancement of conductivity under illumination. Convenient way of fabricating MoS_2_-based FET sensor is expected, which is significant in practical applications [25]. In addition, Lee et al. fabricated the sensor without dielectric layer on multilayer MoS_2_ FETs, which possess hydrophobic interface that serves as novel non-dielectric layer, thus resulting in the improvement of sensitivity [93]. With the nature of 2D structure, MoS_2_-based FET sensors should be studied further to explore higher sensitivity, lower cost, more effective biosensors, and chemical gas sensors, which are potential for next-generation medical diagnosis of cancer, environment monitoring, and food safety.

### Applications of MoS_2_-Based FETs in Memory Devices

**Fig. 10:**
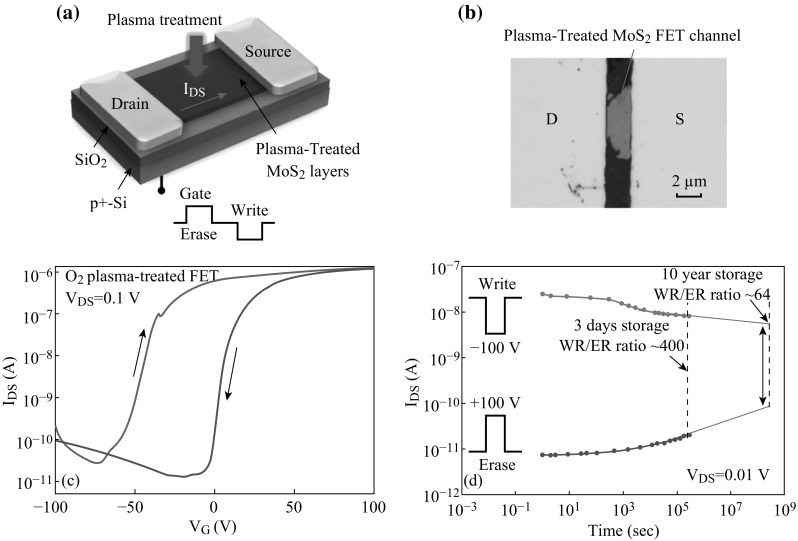
**a** The schematic diagram of plasma-treated MoS_2_ FET. **b** The optical image of as-treated MoS_2_ FET. **c** Transfer characteristic curve (*I*_ds_ vs. *V*_G_). **d** Retention measurements of this FET. Adopted from [97]

Multibit memory devices have attracted much attention and investigations, which are generally fabricated by organic semiconductor materials [94], nanostructure materials [95], and phase-change materials [96]. It is significant to explore a novel convenient method to fabricate multibit memory. Toward this effort, Chen et al. [97] have explored an approach to fabricated MoS_2_ FET-based 2–4 bit memory devices. They proposed a plasma-treated way and found that this plasma-treated MoS_2_ FET could act as multibit memory devices as illustrated in Fig. 10. Figure 10a is the schematic diagram of plasma-treated MoS_2_ FET, of which a 15–30-nm MoS_2_ film served as the active layer with 5 nm Ti/50 nm Au electrode. The optical image of as-treated MoS_2_ FET is shown in Fig. 10b. Herein, D and S are the Ti electrode and Au electrode, respectively. Figure 10c shows the transport characteristic curve (*I*_DS_ (*I*_ds_) vs. *V*_G_); in addition, the measurements of retention are given in Fig. 10d, and it can be seen that the write/read ratio is about 10^3^ after 1 h and 400 after 3 days. Accordingly, a write/read ratio value of about 64 of the as-fabricated FET aged after 10 years can be inferred, which is still valid for circuit application. It is further found that the plasma-treated MoS_2_ FET is faster in programing than the untreated one in their experiments. A physical model for explaining the performance enhancement was proposed as following: The plasma-treated channel top layer could be separated and forms an ambipolar charge-trapping layer, allowing the high-performance non-volatile retention and multibit states in this FET. This method is certainly worth considering for fabrication of nanodevices since it is technically convenient and provides a relatively simple way for realizing non-volatile memory devices, which also offers an effective method to scale down current circuit in future nanoelectronics.

**Fig. 11:**
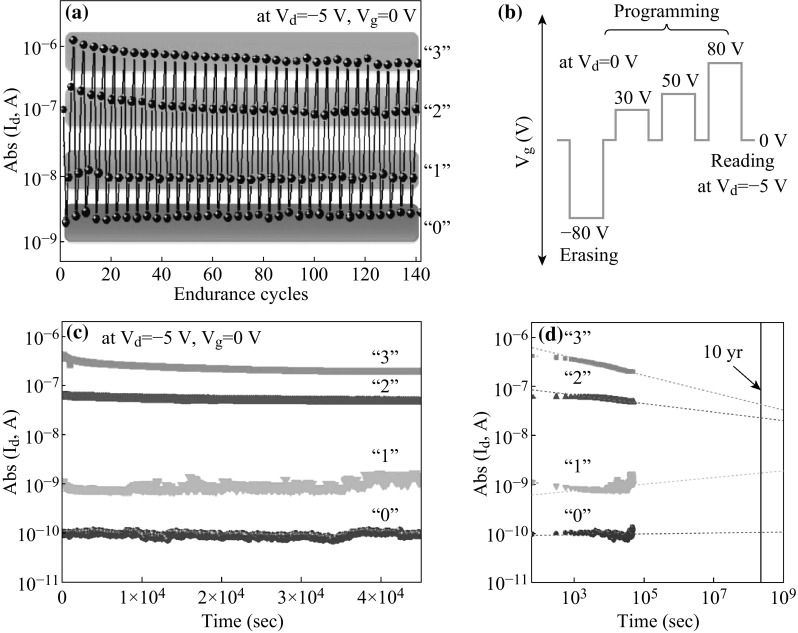
**a** Endurance measurement of as-fabricated NFGM under the condition of *V*_d_ = −5 V and V_g_ = 0 V. **b** Programming process and corresponding voltages are shown, four voltage steps are −80, +30, +50, and +80 V. **c** and **d** illustrate the retention characteristics of four current levels at *V*_d_ = −5 V and *V*_*g*_ = 0 V with 60 s delay. Adopted from [98]

Moreover, MoS_2_ nanoflakes have been employed as charge-trapping layer (nano-floating gate) to fabricate organic nano-floating gate memories (NFGMs) by Kang et al. [98], which are based on organic (poly (3-hexylthiophene) (P3HT)) FET. The inserted solution process is convenient and realized at low temperature to introduce MoS_2_ nanoflakes between two dielectric layers: polystyrene (PS) and poly (methyl methacrylate) (PMMA). The as-fabricated memory device exhibits multilevel non-volatile memory nature, as illustrated in Fig. 11. Figure 11a depicts the endurance of this NFGM, which is more than 10^2^ times (under the condition of *V*_d_ = −5 V and *V*_g_ = 0 V). Programing process and corresponding voltages are shown in Fig. 11b, and the four voltage steps are from −80 to 80 V (−80, +30, +50, +80 V). Figure 11c, d illustrates the retention characteristics by measuring four current levels (Abs (*I*_d_) represents the absolute value of drain current) at *V*_d_ = −5 V and *V*_g_ = 0 V with 60 s delay, indicating the stable retention times after 10 years. This MoS_2_-based organic FET is a 2-bit memory device controlled by *V* and potential for inexpensive memory devices.

## Conclusions

In this paper, we have reviewed state-of-the-art approaches in MoS_2_ FETs, such as progresses on manufacturing of MoS_2_ FETs, MoS_2_ FET-based memory devices, and MoS_2_ FET-based sensors. To understanding the contact physics based on Schottky barrier, different species of metals utilized to achieve high-performance n-type and p-type MoS_2_ FETs are reviewed, and optimization of ferromagnetic contact for spintronics applications are discussed too. Intrinsic properties measured by four-terminal measurements are highlighted, which is an effective method to estimate contact quality of MoS_2_-based FETs. In addition, gate dielectric formation and doping strategies are studied and provide guidelines for prospective manufacturing of MoS_2_-based FETs.

Low-frequency noise (LFN) analysis was carried out for studying the performance of MoS_2_ FETs. FETs made of bilayer MoS_2_ present a longer trap decay time. Further analysis shows that the LFN subjects not only to the physical properties of the channels but also the behavior of contacts in MoS_2_ FETs. The noise increase in aged MoS_2_ FETs is caused by aging of the MoS_2_ channels rather than the aging of contacts. This phenomenon is significant in MoS_2_ as well as in other 2D materials FETs for the optimization of channel implementation.

Photoresponse of MoS_2_-based FETs are critical and considered in this review, mainly focusing on the photocurrent generation with and without illumination. Moreover, MoS_2_-based FETs are utilized in gas and biological sensors, showing its high sensitivity and selectivity. MoS_2_ nanoflakes are fabricated and successfully employed in organic nano-floating gate memories (NFGMs) as non-volatile random-access memory (NVRAM), providing an instance for nanomaterials used in memory devices. The plasma-treated MoS_2_ FETs can serve as multibit memory devices and exhibit excellent storage capacities, suggesting the significance of plasma in performance improving of MoS_2_ electronic devices.

To conclude, MoS_2_ FETs based on thin-film and nano-size structures are investigated. Some key optical and electronic properties of these MoS_2_ FET devices are unique and superior than FETs made of conventional semiconductors, thus are suitable for novel electronic and optoelectronic applications.
